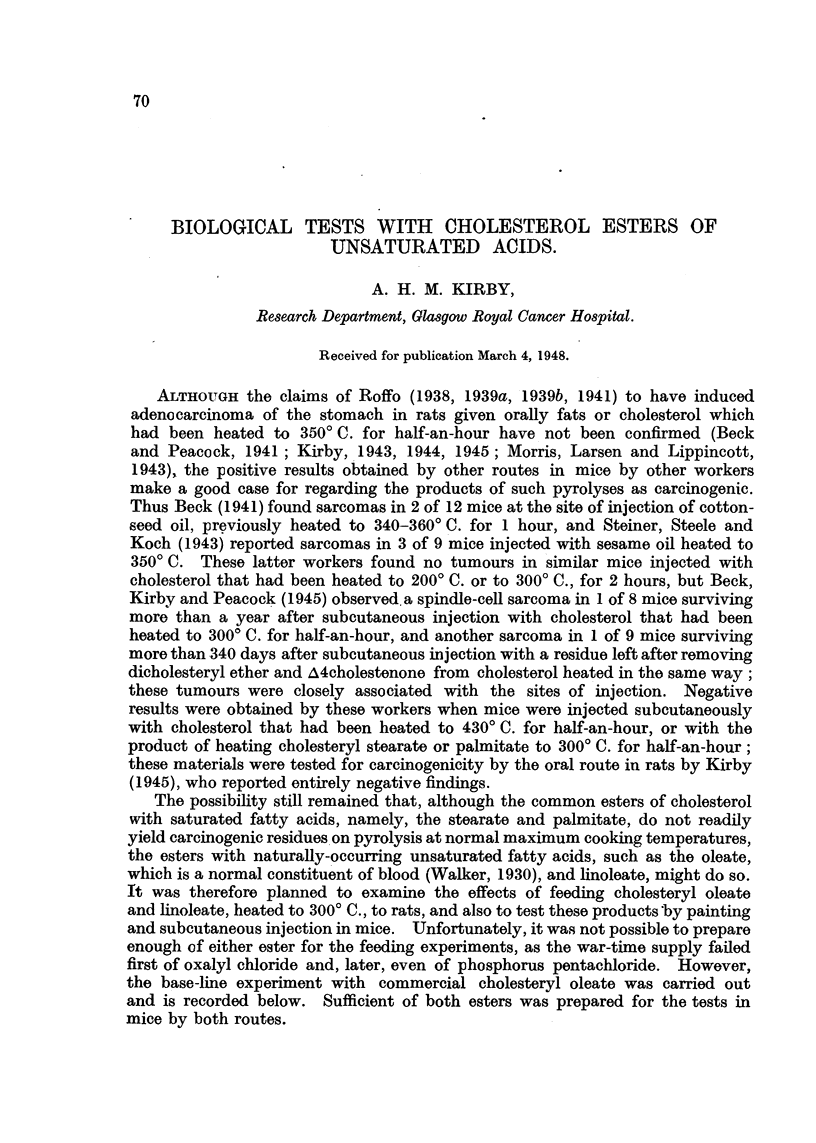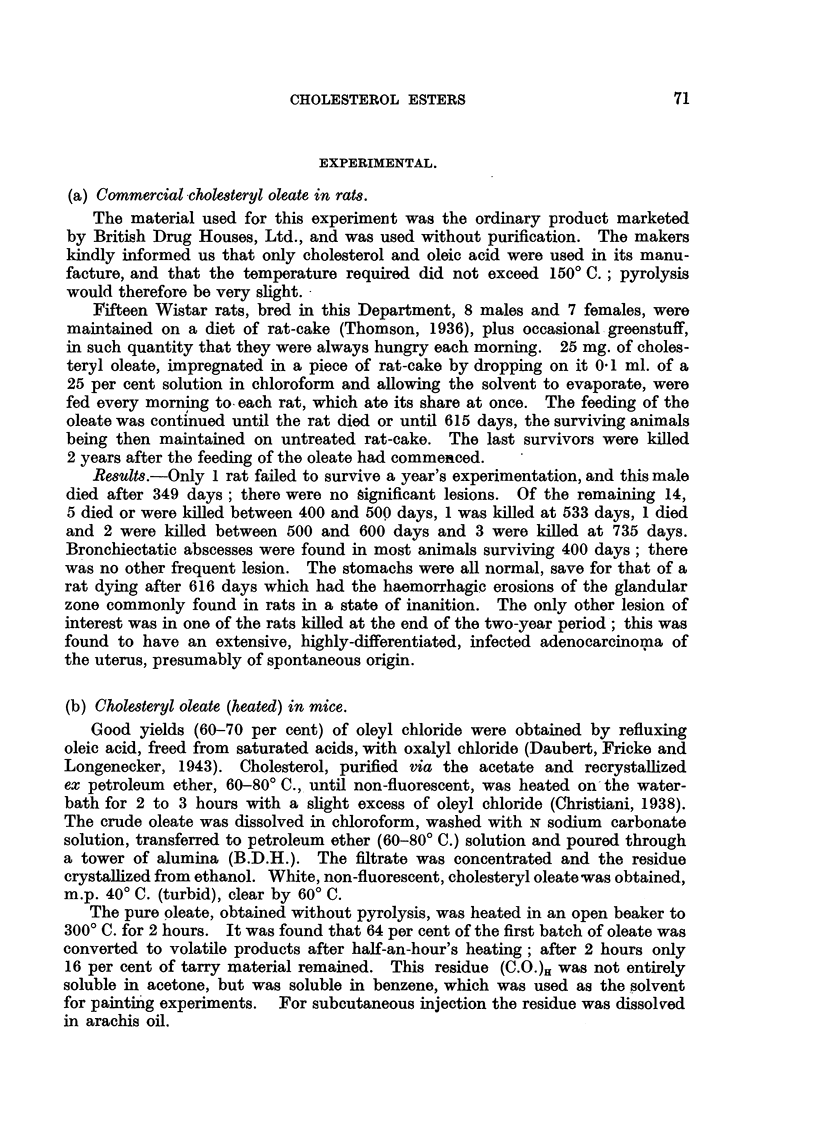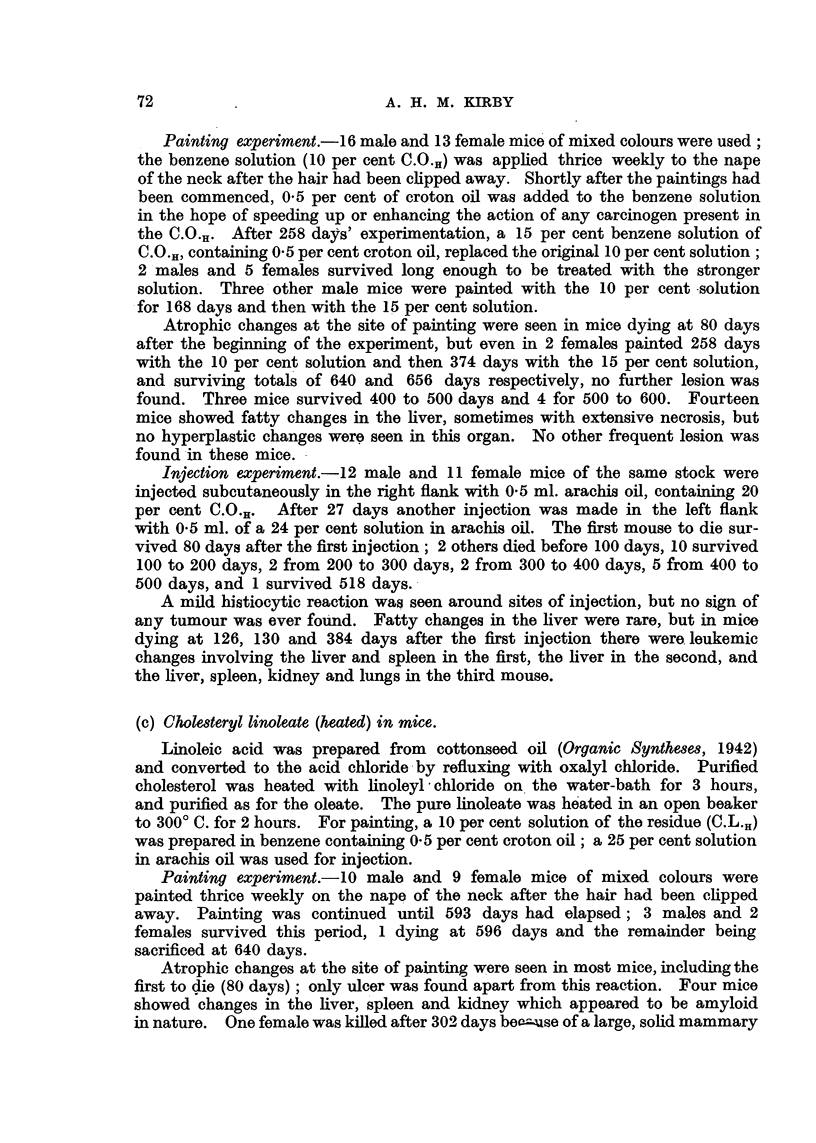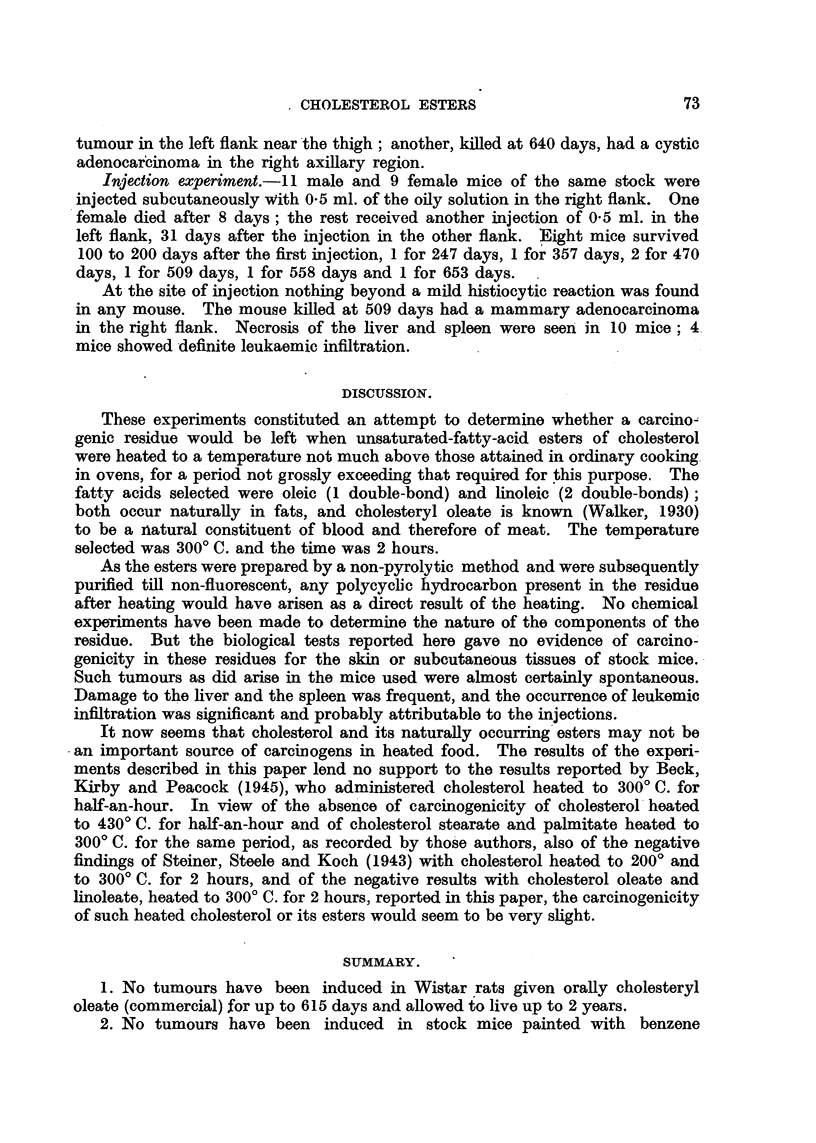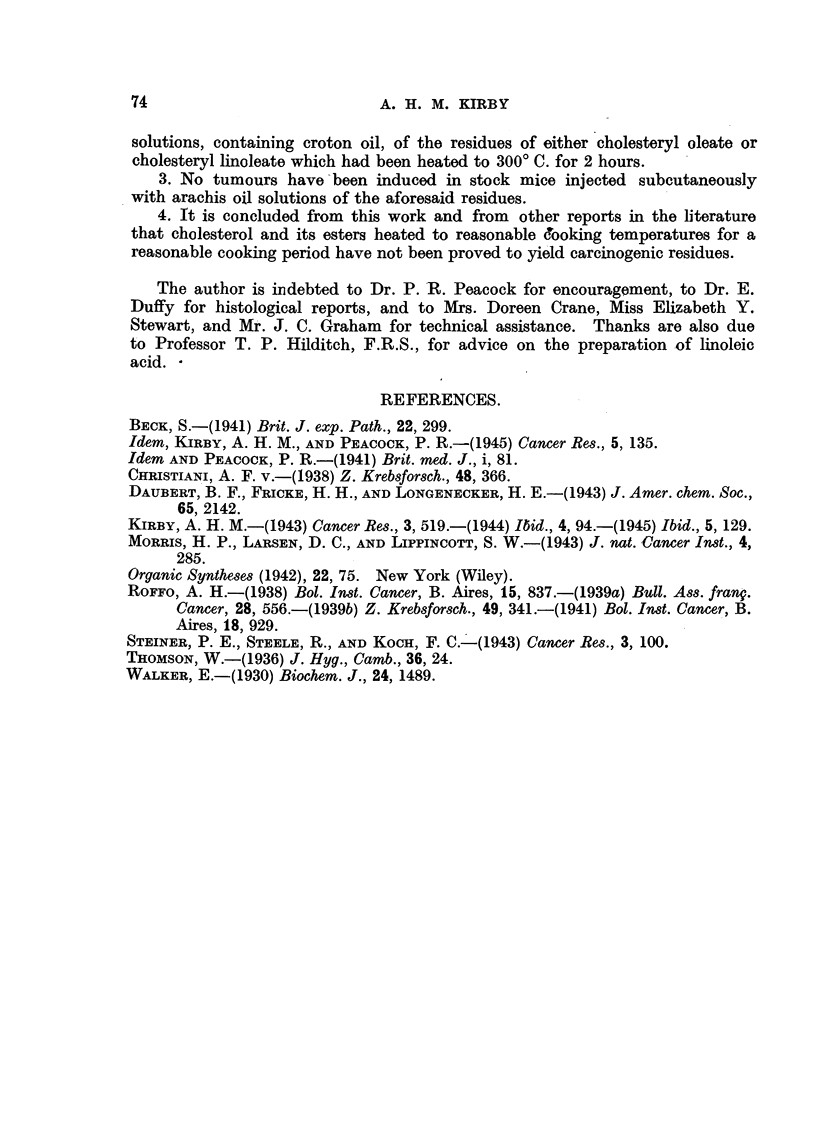# Biological Tests with Cholesterol Esters of Unsaturated Acids

**DOI:** 10.1038/bjc.1948.9

**Published:** 1948-03

**Authors:** A. H. M. Kirby


### BIOLOGICAL TESTS WITH CHOLESTEROL ESTERS OF UNSATURATED ACIDS.

A. H. M. KIRBY,

*Research Department, Glasgow Royal Cancer Hospital.*

Received for publication March 4, 1948.

ALTHOUGH the claims of Roffo (1938, 1939*a*, 1939*b*, 1941) to have induced adenocarcinoma of the stomach in rats given orally fats or cholesterol which had been heated to 350° C. for half-an-hour have not been confirmed (Beck and Peacock, 1941 ; Kirby, 1943, 1944, 1945 ; Morris, Larsen and Lippincott, 1943), the positive results obtained by other routes in mice by other workers make a good case for regarding the products of such pyrolyses as carcinogenic. Thus Beck (1941) found sarcomas in 2 of 12 mice at the site of injection of cotton-seed oil, previously heated to 340–360° C. for 1 hour, and Steiner, Steele and Koch (1943) reported sarcomas in 3 of 9 mice injected with sesame oil heated to 350° C. These latter workers found no tumours in similar mice injected with cholesterol that had been heated to 200° C. or to 300° C., for 2 hours, but Beck, Kirby and Peacock (1945) observed a spindle-cell sarcoma in 1 of 8 mice surviving more than a year after subcutaneous injection with cholesterol that had been heated to 300° C. for half-an-hour, and another sarcoma in 1 of 9 mice surviving more than 340 days after subcutaneous injection with a residue left after removing dicholesteryl ether and Δ4cholestenone from cholesterol heated in the same way ; these tumours were closely associated with the sites of injection. Negative results were obtained by these workers when mice were injected subcutaneously with cholesterol that had been heated to 430° C. for half-an-hour, or with the product of heating cholesteryl stearate or palmitate to 300° C. for half-an-hour ; these materials were tested for carcinogenicity by the oral route in rats by Kirby (1945), who reported entirely negative findings.

The possibility still remained that, although the common esters of cholesterol with saturated fatty acids, namely, the stearate and palmitate, do not readily yield carcinogenic residues on pyrolysis at normal maximum cooking temperatures, the esters with naturally-occurring unsaturated fatty acids, such as the oleate, which is a normal constituent of blood (Walker, 1930), and linoleate, might do so. It was therefore planned to examine the effects of feeding cholesteryl oleate and linoleate, heated to 300° C., to rats, and also to test these products by painting and subcutaneous injection in mice. Unfortunately, it was not possible to prepare enough of either ester for the feeding experiments, as the war-time supply failed first of oxalyl chloride and, later, even of phosphorus pentachloride. However, the base-line experiment with commercial cholesteryl oleate was carried out and is recorded below. Sufficient of both esters was prepared for the tests in mice by both routes.

### EXPERIMENTAL.

(a) *Commercial cholesteryl oleate in rats.*

The material used for this experiment was the ordinary product marketed by British Drug Houses, Ltd., and was used without purification. The makers kindly informed us that only cholesterol and oleic acid were used in its manufacture, and that the temperature required did not exceed 150° C.; pyrolysis would therefore be very slight.

Fifteen Wistar rats, bred in this Department, 8 males and 7 females, were maintained on a diet of rat-cake (Thomson, 1936), plus occasional greenstuff, in such quantity that they were always hungry each morning. 25 mg. of cholesteryl oleate, impregnated in a piece of rat-cake by dropping on it 0·1 ml. of a 25 per cent solution in chloroform and allowing the solvent to evaporate, were fed every morning to each rat, which ate its share at once. The feeding of the oleate was continued until the rat died or until 615 days, the surviving animals being then maintained on untreated rat-cake. The last survivors were killed 2 years after the feeding of the oleate had commenced.

*Results.*—Only 1 rat failed to survive a year's experimentation, and this male died after 349 days; there were no significant lesions. Of the remaining 14, 5 died or were killed between 400 and 500 days, 1 was killed at 533 days, 1 died and 2 were killed between 500 and 600 days and 3 were killed at 735 days. Bronchiectatic abscesses were found in most animals surviving 400 days; there was no other frequent lesion. The stomachs were all normal, save for that of a rat dying after 616 days which had the haemorrhagic erosions of the glandular zone commonly found in rats in a state of inanition. The only other lesion of interest was in one of the rats killed at the end of the two-year period; this was found to have an extensive, highly-differentiated, infected adenocarcinoma of the uterus, presumably of spontaneous origin.

(b) *Cholesteryl oleate (heated) in mice.*

Good yields (60–70 per cent) of oleyl chloride were obtained by refluxing oleic acid, freed from saturated acids, with oxalyl chloride (Daubert, Fricke and Longenecker, 1943). Cholesterol, purified *via* the acetate and recrystallized *ex* petroleum ether, 60–80° C., until non-fluorescent, was heated on the water-bath for 2 to 3 hours with a slight excess of oleyl chloride (Christiani, 1938). The crude oleate was dissolved in chloroform, washed with N sodium carbonate solution, transferred to petroleum ether (60–80° C.) solution and poured through a tower of alumina (B.D.H.). The filtrate was concentrated and the residue crystallized from ethanol. White, non-fluorescent, cholesteryl oleate was obtained, m.p. 40° C. (turbid), clear by 60° C.

The pure oleate, obtained without pyrolysis, was heated in an open beaker to 300° C. for 2 hours. It was found that 64 per cent of the first batch of oleate was converted to volatile products after half-an-hour's heating; after 2 hours only 16 per cent of tarry material remained. This residue $(C.O.)_H$ was not entirely soluble in acetone, but was soluble in benzene, which was used as the solvent for painting experiments. For subcutaneous injection the residue was dissolved in arachis oil.

*Painting experiment.*—16 male and 13 female mice of mixed colours were used; the benzene solution (10 per cent $C.O._H$) was applied thrice weekly to the nape of the neck after the hair had been clipped away. Shortly after the paintings had been commenced, 0·5 per cent of croton oil was added to the benzene solution in the hope of speeding up or enhancing the action of any carcinogen present in the $C.O._H$. After 258 days' experimentation, a 15 per cent benzene solution of $C.O._H$, containing 0·5 per cent croton oil, replaced the original 10 per cent solution; 2 males and 5 females survived long enough to be treated with the stronger solution. Three other male mice were painted with the 10 per cent solution for 168 days and then with the 15 per cent solution.

Atrophic changes at the site of painting were seen in mice dying at 80 days after the beginning of the experiment, but even in 2 females painted 258 days with the 10 per cent solution and then 374 days with the 15 per cent solution, and surviving totals of 640 and 656 days respectively, no further lesion was found. Three mice survived 400 to 500 days and 4 for 500 to 600. Fourteen mice showed fatty changes in the liver, sometimes with extensive necrosis, but no hyperplastic changes were seen in this organ. No other frequent lesion was found in these mice.

*Injection experiment.*—12 male and 11 female mice of the same stock were injected subcutaneously in the right flank with 0·5 ml. arachis oil, containing 20 per cent $C.O._H$. After 27 days another injection was made in the left flank with 0·5 ml. of a 24 per cent solution in arachis oil. The first mouse to die survived 80 days after the first injection; 2 others died before 100 days, 10 survived 100 to 200 days, 2 from 200 to 300 days, 2 from 300 to 400 days, 5 from 400 to 500 days, and 1 survived 518 days.

A mild histiocytic reaction was seen around sites of injection, but no sign of any tumour was ever found. Fatty changes in the liver were rare, but in mice dying at 126, 130 and 384 days after the first injection there were leukemic changes involving the liver and spleen in the first, the liver in the second, and the liver, spleen, kidney and lungs in the third mouse.

(c) *Cholesteryl linoleate (heated) in mice.*

Linoleic acid was prepared from cottonseed oil (*Organic Syntheses*, 1942) and converted to the acid chloride by refluxing with oxalyl chloride. Purified cholesterol was heated with linoleyl chloride on the water-bath for 3 hours, and purified as for the oleate. The pure linoleate was heated in an open beaker to 300° C. for 2 hours. For painting, a 10 per cent solution of the residue ($C.L._H$) was prepared in benzene containing 0·5 per cent croton oil; a 25 per cent solution in arachis oil was used for injection.

*Painting experiment.*—10 male and 9 female mice of mixed colours were painted thrice weekly on the nape of the neck after the hair had been clipped away. Painting was continued until 593 days had elapsed; 3 males and 2 females survived this period, 1 dying at 596 days and the remainder being sacrificed at 640 days.

Atrophic changes at the site of painting were seen in most mice, including the first to die (80 days); only ulcer was found apart from this reaction. Four mice showed changes in the liver, spleen and kidney which appeared to be amyloid in nature. One female was killed after 302 days because of a large, solid mammary tumour in the left flank near the thigh ; another, killed at 640 days, had a cystic adenocarcinoma in the right axillary region.

*Injection experiment.*—11 male and 9 female mice of the same stock were injected subcutaneously with 0·5 ml. of the oily solution in the right flank. One female died after 8 days ; the rest received another injection of 0·5 ml. in the left flank, 31 days after the injection in the other flank. Eight mice survived 100 to 200 days after the first injection, 1 for 247 days, 1 for 357 days, 2 for 470 days, 1 for 509 days, 1 for 558 days and 1 for 653 days.

At the site of injection nothing beyond a mild histiocytic reaction was found in any mouse. The mouse killed at 509 days had a mammary adenocarcinoma in the right flank. Necrosis of the liver and spleen were seen in 10 mice ; 4 mice showed definite leukaemic infiltration.

### DISCUSSION.

These experiments constituted an attempt to determine whether a carcinogenic residue would be left when unsaturated-fatty-acid esters of cholesterol were heated to a temperature not much above those attained in ordinary cooking in ovens, for a period not grossly exceeding that required for this purpose. The fatty acids selected were oleic (1 double-bond) and linoleic (2 double-bonds) ; both occur naturally in fats, and cholesteryl oleate is known (Walker, 1930) to be a natural constituent of blood and therefore of meat. The temperature selected was 300° C. and the time was 2 hours.

As the esters were prepared by a non-pyrolytic method and were subsequently purified till non-fluorescent, any polycyclic hydrocarbon present in the residue after heating would have arisen as a direct result of the heating. No chemical experiments have been made to determine the nature of the components of the residue. But the biological tests reported here gave no evidence of carcinogenicity in these residues for the skin or subcutaneous tissues of stock mice. Such tumours as did arise in the mice used were almost certainly spontaneous. Damage to the liver and the spleen was frequent, and the occurrence of leukemic infiltration was significant and probably attributable to the injections.

It now seems that cholesterol and its naturally occurring esters may not be an important source of carcinogens in heated food. The results of the experiments described in this paper lend no support to the results reported by Beck, Kirby and Peacock (1945), who administered cholesterol heated to 300° C. for half-an-hour. In view of the absence of carcinogenicity of cholesterol heated to 430° C. for half-an-hour and of cholesterol stearate and palmitate heated to 300° C. for the same period, as recorded by those authors, also of the negative findings of Steiner, Steele and Koch (1943) with cholesterol heated to 200° and to 300° C. for 2 hours, and of the negative results with cholesterol oleate and linoleate, heated to 300° C. for 2 hours, reported in this paper, the carcinogenicity of such heated cholesterol or its esters would seem to be very slight.

### SUMMARY.

1. No tumours have been induced in Wistar rats given orally cholesteryl oleate (commercial) for up to 615 days and allowed to live up to 2 years.

2. No tumours have been induced in stock mice painted with benzene solutions, containing croton oil, of the residues of either cholesteryl oleate or cholesteryl linoleate which had been heated to 300° C. for 2 hours.

3. No tumours have been induced in stock mice injected subcutaneously with arachis oil solutions of the aforesaid residues.

4. It is concluded from this work and from other reports in the literature that cholesterol and its esters heated to reasonable cooking temperatures for a reasonable cooking period have not been proved to yield carcinogenic residues.

The author is indebted to Dr. P. R. Peacock for encouragement, to Dr. E. Duffy for histological reports, and to Mrs. Doreen Crane, Miss Elizabeth Y. Stewart, and Mr. J. C. Graham for technical assistance. Thanks are also due to Professor T. P. Hilditch, F.R.S., for advice on the preparation of linoleic acid.

### REFERENCES.

Beck, S.—(1941) *Brit. J. exp. Path.*, **22**, 299.
*Idem*, Kirby, A. H. M., and Peacock, P. R.—(1945) *Cancer Res.*, **5**, 135.
*Idem* and Peacock, P. R.—(1941) *Brit. med. J.*, i, 81.
Christiani, A. F. v.—(1938) *Z. Krebsforsch.*, **48**, 366.
Daubert, B. F., Fricke, H. H., and Longenecker, H. E.—(1943) *J. Amer. chem. Soc.*, **65**, 2142.
Kirby, A. H. M.—(1943) *Cancer Res.*, **3**, 519.—(1944) *Ibid.*, **4**, 94.—(1945) *Ibid.*, **5**, 129.
Morris, H. P., Larsen, D. C., and Lippincott, S. W.—(1943) *J. nat. Cancer Inst.*, **4**, 285.
*Organic Syntheses* (1942), **22**, 75. New York (Wiley).
Roffo, A. H.—(1938) *Bol. Inst. Cancer*, B. Aires, **15**, 837.—(1939*a*) *Bull. Ass. franç. Cancer*, **28**, 556.—(1939*b*) *Z. Krebsforsch.*, **49**, 341.—(1941) *Bol. Inst. Cancer*, B. Aires, **18**, 929.
Steiner, P. E., Steele, R., and Koch, F. C.—(1943) *Cancer Res.*, **3**, 100.
Thomson, W.—(1936) *J. Hyg., Camb.*, **36**, 24.
Walker, E.—(1930) *Biochem. J.*, **24**, 1489.